# Supplementary material for: What does it take to learn the rules of RNA base pairing? A lot less than you may think
Source: bioRxiv. 2025 Aug 2:2025.07.31.668042. Preprint. [Version 1] doi: 10.1101/2025.07.31.668042 (PMC12324431; doi:10.1101/2025.07.31.668042)
Supplement: Supplement 1 [file media-1.gz › supplemental_material/src/rscape_v2.6.0.tar.gz!/rscape_v2.6.0/lib/infernal/Manuscripts/filter_thresholds/figs/SRP_fst.pdf]

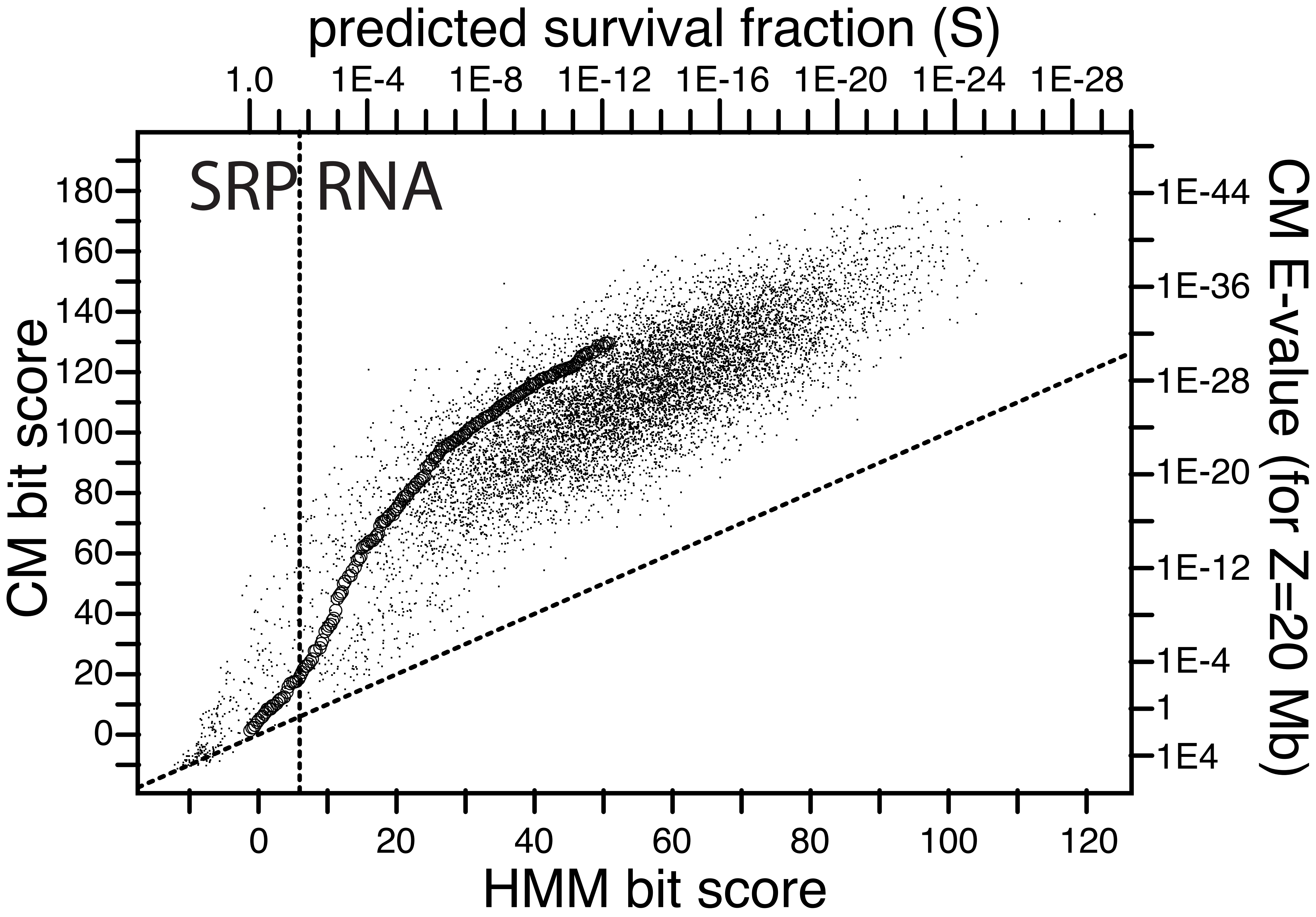

predicted survival fraction (S)
1.0
1E-4
1E-8
1E-12
1E-16
1E-20
1E-24
1E-28
SRP RNA
CM bit score
180
160
140
120
100
80
60
40
20
0
CM E-value (for Z=20 Mb)
1E-44
1E-36
1E-28
1E-20
1E-12
1E-4
1
1E4
0
20
40
60
80
100
120
HMM bit score
